# Long noncoding RNAs Colorectal Neoplasia Differentially Expressed and taurine-upregulated gene 1 are downregulated in sepsis and positively regulate each other to suppress the apoptosis of cardiomyocytes

**DOI:** 10.1080/21655979.2021.2008658

**Published:** 2021-12-07

**Authors:** Zhenwei Xu, Xingyu Lin, Jingfa Zhu, Zhixia Zhu

**Affiliations:** a Department of Emergency, Quanzhou First Hospital Affiliated to Fujian Medical University, Quanzhou City, Fujian Province, PR. China; b Department of Emergency, Fujian Medical University Union Hospital, Fuzhou City, Fujian Province, PR China

**Keywords:** CRNDE, TUG1, cardiomyocytes, sepsis

## Abstract

Long noncoding RNAs (lncRNAs) Colorectal Neoplasia Differentially Expressed (CRNDE) and taurine-upregulated gene 1 (TUG1) play similar roles in sepsis, indicating the existence of the crosstalk between them. Sepsis is a major cause of injuries in heart, which are related to high mortality rates. This study was therefore carried out to analyze the potential crosstalk between CRNDE and TUG1 in sepsis, with a focus on sepsis-induced cell apoptosis in heart. Expression of CRNDE and TUG1 was analyzed with RT-qPCR. Correlations between them were analyzed by Pearson’s correlation coefficient. CRNDE and TUG1 were overexpressed in cardiomyocytes to determine the relationship between them. The roles of CRNDE and TUG1 in regulating the apoptosis of cardiomyocytes were explored by cell apoptosis assay. We found that both CRNDE and TUG1 were downregulated in sepsis. In cardiomyocytes, LPS treatment resulted in the downregulation of CRNDE and TUG1. Overexpression of CRNDE and TUG1 in cardiomyocytes increased the expression levels of each other. Under lipopolysaccharide (LPS) treatment, decreased apoptosis rates of cardiomyocytes were observed after CRNDE and TUG1 overexpression. CRNDE and TUG1 co-overexpression showed a stronger effect. In conclusion, CRNDE and TUG1 are downregulated in sepsis and they positively regulate each other to suppress the apoptosis of cardiomyocytes.

## Introduction

Sepsis is defined as a systemic inflammation-caused severe infections that cause a series of rapid and severe events, such as impaired blood flow and leaking blood vessels [1]. Sepsis is usually caused by bacterial, fungal, and viral infections [2]. Sepsis is usually not recognized by patients until late stages, such as septic shock [3], which is correlated with unacceptable high mortality rate [4]. It is estimated that about 40% of patients with septic shock will die within a short-term after the diagnosis [5]. Patients with mild or moderate sepsis are usually treated with antibiotics [6]. However, in severe cases, even systemic antibiotics treatment fails frequently [6]. Therefore, more effective treatments are still needed.

Sepsis may cause the development of clinical disorders in most organs, such as the heart, leading to cardiomyopathy [7,8]. It has been well established that molecular factors play critical roles in sepsis-induced organ failures [9]. Targeted therapy can be performed to reverse disease progression by modulating gene expression [10,11]. Without direct roles in coding proteins, long noncoding RNAs (lncRNAs) regulate diseases, such as sepsis, by indirectly affecting gene expression [12,13]. In effect, previous studies have showed that lncRNAs are promising biomarkers for the prediction of sepsis and potential therapeutic targets for sepsis treatment [13]. However, the expression pattern and functions of most lncRNAs in sepsis remain elusive. Previous studies have shown that both lncRNAs Colorectal Neoplasia Differentially Expressed (CRNDE) and taurine-upregulated gene 1 (TUG1) play protective roles in sepsis-induced kidney injury [14,15]. Therefore, it is possible that CRNDE and TUG1 may interact with each other to participate in sepsis. This study was then performed to explore the potential crosstalk between TUG1 and CRNDE in sepsis.

## Materials and Methods

### Participants

From May 2019 to May 2020, this study enrolled a total of 64 sepsis patients (30 females and 34 males) and 64 healthy controls (30 females and 34 males) at Quanzhou First Hospital Affiliated to Fujian Medical University. Ethics Committee of this hospital approved this study. Patients were diagnosed by blood and urine tests. The abnormal level of bacteria blood and urine was observed. Patients’ age ranged from 41 to 68 years (54.8 ± 7.8 years). To match the age distribution of sepsis patients, the age of healthy controls also ranged from 41 to 68 years (54.7 ± 7.7 years). In the 64 patients, sepsis was caused by bacterial infections. No previous history of sepsis and other severe diseases, such as cancers, heart diseases, diabetes and viral infections, was observed in patients. No therapy was initiated prior to this study. Systemic physiological examinations were performed on all healthy controls, and all physiological functions of all controls were within the normal range. All patients and controls signed informed consent.

### Plasma and cardiomyocytes

Prior to therapy, fasting blood (3 ml) was extracted from all patients to prepare samples of plasma. A liquid nitrogen tank was used to store plasma samples before use. The AC16 (Sigma-Aldrich) human cardiomyocyte cell line from Sigma-Aldrich (USA) was used. Cardiomyocyte growth medium (ScienCell Research) was used to cultivate AC16 cells in a 95% humidity and 5% CO_2_ incubator at 37 °C. The subsequent experiments were carried out using cells harvested from passages 3 to 5. In cases of lipopolysaccharide (LPS) treatment, AC16 cells were treated with LPS (0, 1, 2, 5, and 10 μg/ml, Sigma-Aldrich) for 48 h before the subsequent experiments.

### Transient cell transfections

Backbone vector-expressing CRNDE (NCBI Accession: NR_034105.4) or TUG1 (NCBI Accession: NR_130147.2) was constructed with pcDNA3.1 (Invitrogen). The cDNA synthesis service was provided by Invitrogen. Using Lipofectamine 2000 (Invitrogen), 10^8^ AC16 cells were transfected with 1 μg of CRNDE or TUG1 expression vector. Briefly, the vector was first mixed with Lipofectamine 2000 to prepare the transfection mixture, followed by incubating cells with the transfection mixture for 6 h. After that, cells were washed with fresh media and cell culturing at 37 °C was performed for 48 h before carrying out the subsequent assays.

### RNA preparations

Plasma samples from both groups and AC16 cells were subjected to total RNA isolation using RNAzol reagent (Sigma-Aldrich). Incubation with DNase I (Invitrogen) was performed for 2 h at 37°C to completely remove genomic DNAs. A Urea-PAGE gel (6%) was used in RNAs to check the integrity of RNA samples. OD ratios of all RNA samples were determined using a NanoDrop™ 2000 Spectrophotometer (Invitrogen) to analyze RNA purity.

### RT-PCR

Using a SS-III-RT system (Invitrogen), RNA samples with satisfactory quality were used to prepare cDNA samples. After that, cDNA samples were used as a template to perform qPCRs using SYBR Green Master Mix (Bio-Rad) to determine the expression of CRNDE, TUG1, and mRNA of Bax and Bcl-2. The crosstalk control was 18S rRNA. PCRs were performed under following thermal cycling conditions: 1 min at 95°C, then 10s at 95°C, and 50s at 58°C for a total of 40 cycles. Ct values CRNDE or TUG1 were normalized to internal control 18S rRNA using the method of 2^−ΔΔCT^.

### Cell apoptosis assay

After cell wash using PBS (pre-cold), AC16 cells were counted and cultivated in a 6-well plate (10,000 cells per well). LPS was added to the final concentration of 10 μg/ml. After that, cell culturing was performed at 37°C for 48 h. After cell washing using PBS (pre-cold), AC16 cells were resuspended in 0.5 ml of Annexin binding buffer. After that, cells were stained in the dark for 12 min with Annexin V FITC and Propidium iodide (PI) (BioLegend). After that, flow cytometry was performed to analyze apoptotic cells. Annexin V was used to detect cellular apoptosis, while late apoptotic or necrotic cells were detected by PI. The combination of Annexin V and PI was applied to distinguish cells at different apoptotic stages.

### Statistical analysis

GraphPad Prism 6 (GraphPad, USA) was applied to compare data sets and plot images. The unpaired t test (two-tail) was applied to compare two groups. One-way ANOVA Tukey’s test was used from multiple group comparisons. p < 0.05 was deemed statistically significant.

## Results

### Downregulation of CRNDE and TUG1 was observed in sepsis

**Figure 1.**
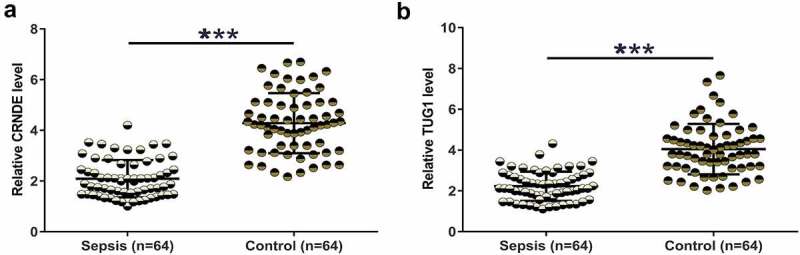
Downregulation of CRNDE and TUG1 was observed in sepsis. Plasma samples from sepsis patients (n = 64) and healthy controls (n = 64) were collected and were subjected to RNA preparations and RT-qPCRs to determine the expression of CRNDE (a) and TUG1 (b). Expression levels of CRNDE and TUG1 in plasma samples from both groups were presented, ***,p < 0.001

Differential expression may suggest the involvement of certain genes in human diseases. To this end, plasma samples were collected and were subjected to RNA preparations and RT-qPCRs to determine the expression of CRNDE and TUG1. Plasma CRNDE was significantly underexpressed in the sepsis group (Figure 1a, p < 0.001). Similarly, TUG1 was also significantly downregulated in the sepsis group (Figure 1b, p < 0.001). It is worth noting that no significant differences in CRNDE and TUG1 expression were observed between males and females (data not shown). Therefore, CRNDE and TUG1 may participate in sepsis.

### CRNDE and TUG1 were positively correlated

**Figure 2.**
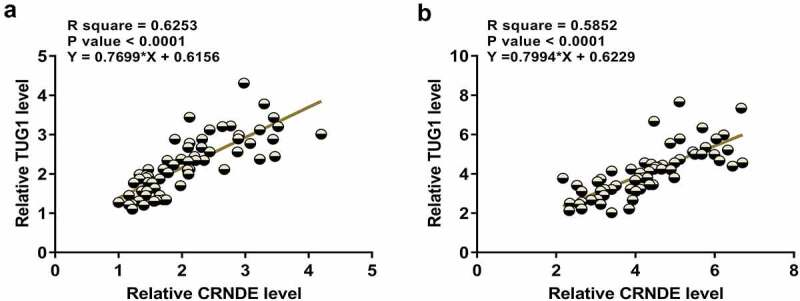
CRNDE and TUG1 were positively correlated. Pearson’s correlation coefficient was calculated to study the correlations between CRNDE and TUG1 across plasma samples from both sepsis patients (a) and healthy controls (b)

Close correlations between two genes may indicate potential interactions. Correlations between CRNDE and TUG1 across plasma samples from both sepsis and control samples were then analyzed. The results showed that CRNDE and TUG1 were positively and significantly correlated across sepsis samples (Figure 2a). Similarly, a significant and positively correlation between CRNDE and TUG1 was also observed across control samples (Figure 2b). The significant correlation between CRNDE and TUG1 may indicate the possible crosstalk between them.

### Overexpression of CRNDE and TUG1 increased the expression of each other in cardiomyocytes with and without LPS treatment

**Figure 3.**
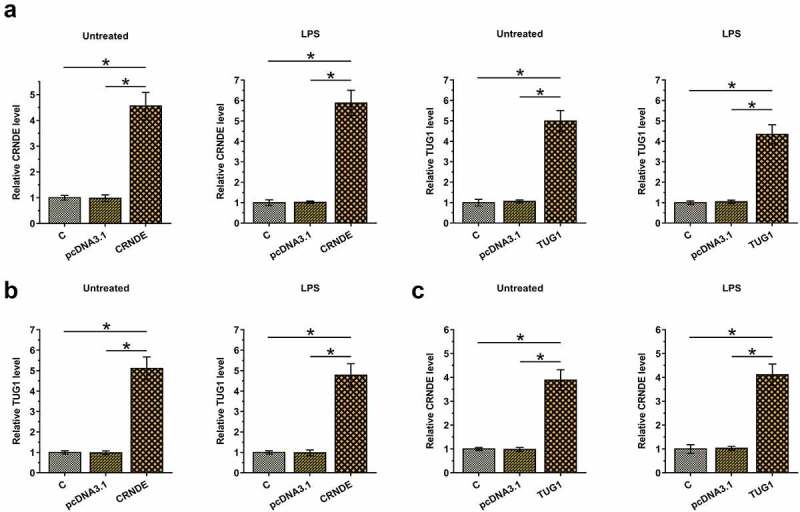
Overexpression of CRNDE and TUG1 increased the expression of each other in cardiomyocytes with and without LPS treatment. To test whether CRNDE and TUG1 can interact with each other, cardiomyocytes with and without LPS treatment (10 μg/ml for 48 h) were overexpressed with CRNDE or TUG1 (a). The effects of CRNDE overexpression on TUG1 (b) and the effects of TUG1 overexpression on CRNDE (c) in both types of cardiomyocytes were also explored by RT-qPCR. *,p < 0.05

The positive correlation between CRNDE and TUG1 may indicate the existence of potential crosstalk between them. To further test whether CRNDE and TUG1 can interact with each other, cardiomyocytes with and without (Untreated group) LPS treatment (10 μg/ml for 48 h) were overexpressed with CRNDE or TUG1 (Figure 3a, p < 0.05). Moreover, overexpression of CRNDE significantly increased the expression of TUG1 (Figure 3b, p < 0.05) and overexpression of TUG1 also significantly increased the expression of CRNDE (Figure 3c, p < 0.05) in both types of cardiomyocytes. Therefore, CRNDE and TUG1 may form a positive feedback loop under both pathological and physiological conditions.

### CRNDE and TUG1 overexpression decreased the apoptosis of cardiomyocytes induced by LPS

**Figure 4.**
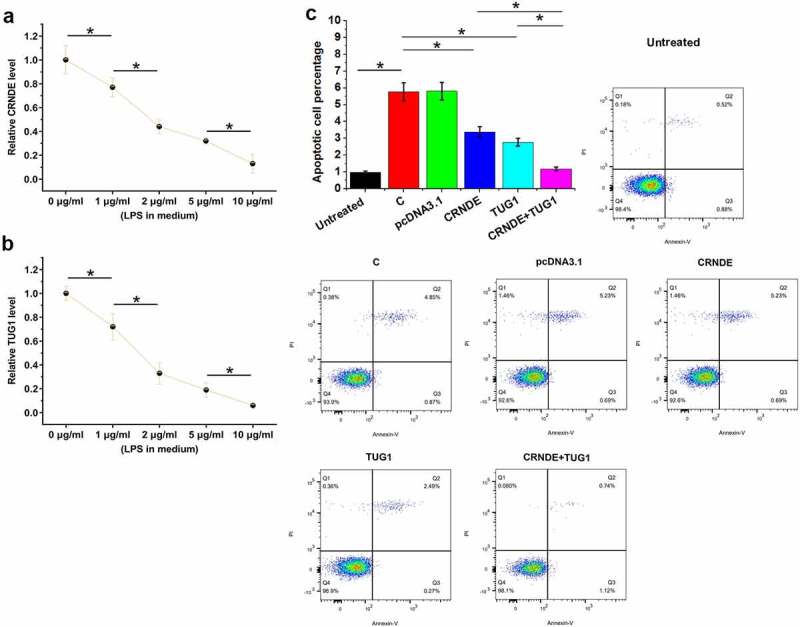
CRNDE and TUG1 overexpression decreased the LPS-induced apoptosis of cardiomyocytes. AC16 cells were subjected to LPS (0, 1, 2, 5, and 10 μg/ml, Sigma-Aldrich) treatment for 48 h, and then CRNDE (a) and TUG1 (b) expression was studied with RT-qPCR. The effects of CRNDE and TUG1 overexpression on the apoptosis of cardiomyocytes treated with LPS (10 μg/ml for 48 h) were analyzed by cell apoptosis assay, and untreated cells were also included (c). *,p < 0.05

Cell apoptosis contributes to sepsis. To test the role of CRNDE and TUG1 in the apoptosis of cardiomyocytes in sepsis, AC16 cells were cultivated in medium supplemented with 0, 1, 2, 5, and 10 μg/ml LPS (Sigma-Aldrich) for 48 h, followed by RT-qPCR to analyze CRNDE and TUG1 expression. LPS treatment significantly decreased the expression of CRNDE (Figure 4a) and TUG1 (Figure 4b) (p < 0.05). The effects of CRNDE and TUG1 overexpression on the apoptosis of cardiomyocytes were analyzed by cell apoptosis assay. Compared to the untreated group (no LPS treatment), increased cell apoptosis was observed in control cells with LPS treatment (C) and pcDNA3.1-transfected cells with LPS treatment (pcDNA3.1). Decreased apoptosis rates of cardiomyocytes were observed after CRNDE and TUG1 overexpression. In addition, the combined overexpression of CRNDE and TUG1 showed a stronger effect (Figure 4c, p < 0.05). Interestingly, the apoptotic rate of the cotransfection group is close to that if the untreated group. Bax/Bcl-2 ratios of all groups were calculated. A similar trend was observed. Therefore, CRNDE and TUG1 may postively regulate each other to inhibit cell apoptosis in sepsis.

## Discussion

This study mainly investigated the crosstalk between CRNDE and TUG1 in sepsis. We found that both CRNDE and TUG1 were downregulated in sepsis and they could form a positive feedback loop to suppress the apoptosis of cardiomyocytes induced by LPS.

Wang et al. showed that CRNDE was significantly downregulated in the rat and cell model of sepsis [14]. CRNDE decreased miR-181a-5p to aggregate sepsis-induced kidney injury [14]. To date, the involvement of CRNDE in sepsis patients is still unknown. We showed that CRNDE was also significantly downregulated in sepsis patients. LPS-induced inflammation plays critical roles in the progression of sepsis. In this study, LPS treatment decreased the expression of CRNDE in cardiomyocytes in a dose-dependent manner and overexpression of CRNDE decreased the apoptosis of cardiomyocytes treated with LPS. Therefore, CRNDE may participate in sepsis through the LPS-dependent pathway to play protective roles.

TUG1 has also been proven to be a critical player in sepsis-induced kidney injury [15]. It was observed that TUG1 was downregulated in sepsis and overexpression of TUG1 targets the axis of miR-142-3p/sirtuin 1 and regulates the NF-kB pathway to suppress disease progression. Consistently, our study also reported the downregulation of TUG1 in sepsis. We showed that TUG1 could also play protective roles in LPS-induced apoptosis of cardiomyocytes, suggesting that TUG1 may participate in multiple sepsis-induced organ failures.

We observed the expression patterns of CRNDE and TUG1 in sepsis patients and their similar roles in the regulation of LPS-induced apoptosis of cardiomyocytes, suggesting possible crosstalk between them. We, in this study, showed that CRNDE and TUG1 could form a positive feedback loop in cardiomyocytes under both pathological (LPS treatment) and physiological (no LPS treatment) conditions. Although we failed to explore the in vivo crosstalk between them, we showed that CRNDE and TUG1 were positively correlated across plasma samples from both sepsis patients and healthy controls, suggesting the existence of the positive feedback loop in both sepsis patients and healthy controls. Although previous studies have reported the involvement of lncRNAs in sepsis [16,17], the crosstalk between lncRNAs and the possible mechanisms have not been well studied. The mechanism that mediates the crosstalk between them (CRNDE and TUG1 for our research) remains unclear. Future studies are still needed.

### Conclusions

**Figure 5.**
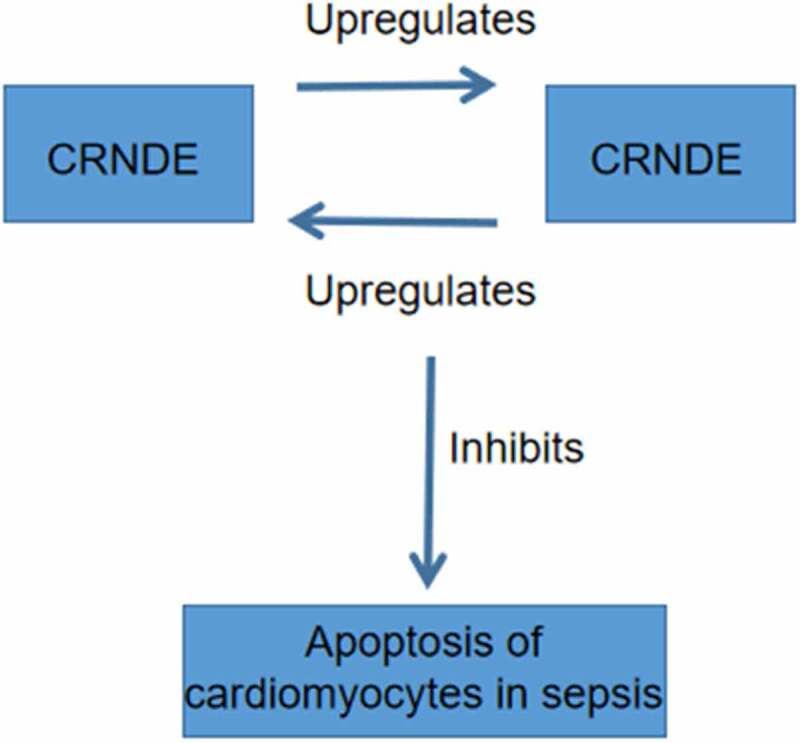
The main finding of the present study

In conclusion, both CRNDE and TUG1 are downregulated in sepsis and they may form a positive feedback loop to suppress the apoptosis of cardiomyocytes induced by LPS (Figure 5).

## Supplementary Material

Supplemental Material

## Data Availability

The data used to support the findings of this study are included within the article.

## Research highlights

1. CRNDE and TUG1 expression was altered in sepsis;
2. CRNDE and TUG1 positively regulate each other;
3. CRNDE and TUG1 suppress the apoptosis of cardiomyocytes in sepsis.
