# Supplementary figures and images for: Long noncoding RNAs Colorectal Neoplasia Differentially Expressed and taurine-upregulated gene 1 are downregulated in sepsis and positively regulate each other to suppress the apoptosis of cardiomyocytes
Source: Bioengineered. 2021 Dec 7;12(2):11369–75. doi: 10.1080/21655979.2021.2008658 (PMC8810183; doi:10.1080/21655979.2021.2008658)

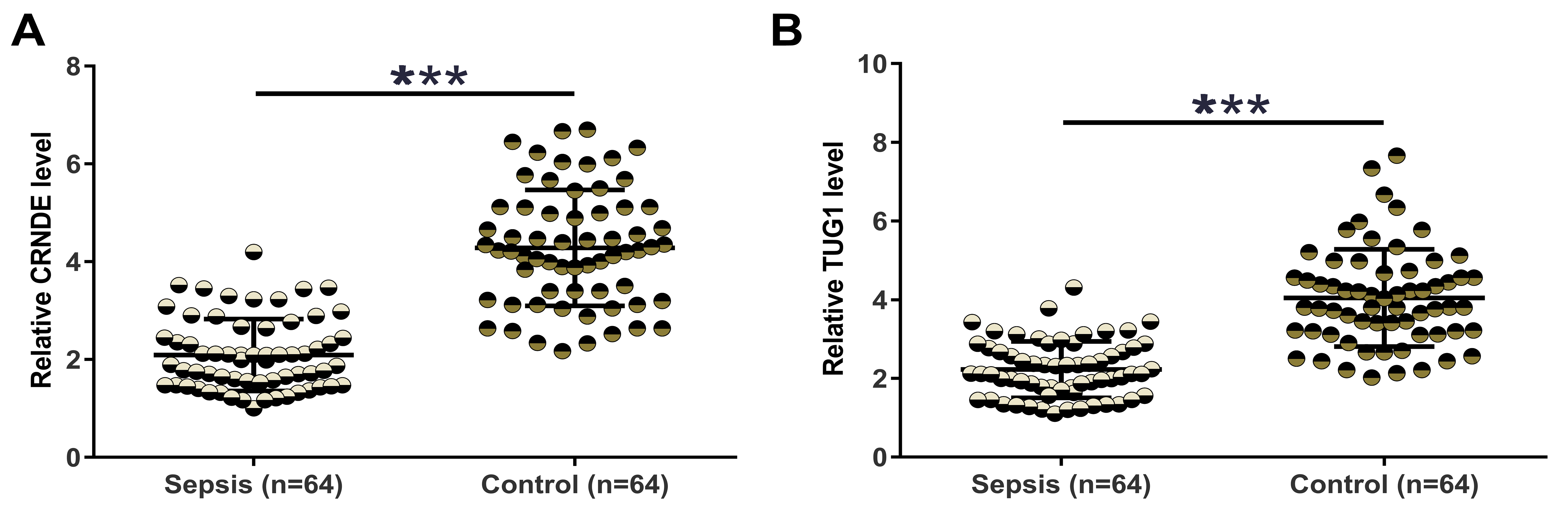

Supplement: Supplemental Material [file KBIE_A_2008658_SM4338.zip › supplementary/Supplemental Figure 1.tif]
